# Corticosteroid Therapy in Severe Cases of Pneumonia Caused by SARS-CoV-2

**DOI:** 10.7759/cureus.33076

**Published:** 2022-12-29

**Authors:** João Alves, Andrea Salgueiro, João Pedro Baptista, Paulo Martins

**Affiliations:** 1 Intensive Care Unit, Centro Hospitalar Universitário de Coimbra, Coimbra, PRT

**Keywords:** sars-cov-2, methylprednisolone, corticotherapy, covid-19, pneumonia

## Abstract

We present a case of severe pneumonia caused by severe acute respiratory syndrome coronavirus 2 (SARS-CoV-2) in a 63-year-old woman needing venous oxygenation by an extracorporeal membrane. Given the difficult clinical resolution with persistent inflammatory parameters, treatment with corticosteroids (methylprednisolone) was prescribed. The clinical evolution observed, namely the improvement of respiratory and imaging parameters, reiterates the recommendations of corticosteroids for moderate to severe disease.

## Introduction

Severe pneumonia caused by severe acute respiratory syndrome coronavirus 2 (SARS-CoV-2) results from a dysregulation of the inflammatory response of the host to SARS-CoV-2 and may phenotypically translate into an early stage of the disease (stage I) in activation of the adaptive immune response with pulmonary involvement (stage II), or in a hyperinflammatory phase associated with worsening of the pulmonary condition and potentially fatal multiorgan failure (stage III) [1]. In this last scenario, as described in the present case report, in addition to supportive therapy, there is a pathophysiological rationale for immunomodulatory therapy, namely corticosteroid therapy. The hyperinflammatory phase of the viral infection is severe at the cellular level, with an increase of the alveolar membrane permeability, which can change the pulmonary perfusion, that is why the inflammatory suppression can have a benefit at the pulmonary and systemic level [2].

On the other hand, immunosuppressive therapy is associated with a greater predisposition to infection, prolonged viral clearance, and other complications such as difficulty in glycemic control, electrolyte disturbances, gastrointestinal bleeding, avascular bone necrosis, delirium, myopathy, and increased mortality [3]. Corticosteroid therapy is recommended for the treatment of SARS-CoV-2-caused severe pneumonia in a patient under extracorporeal membrane oxygenation (ECMO) with associated high mortality [4,5]. For patients with coronavirus disease 2019 (COVID-19) in the need of venovenous oxygenation by an ECMO, corticosteroid therapy is recommended in refractory septic shock or severe acute respiratory distress syndrome (ARDS) [6].

A recovery study demonstrated the benefit of the early use of dexamethasone in patients with hypoxemic SARS-CoV-2 pneumonia requiring oxygen supplementation or those undergoing mechanical ventilation, with a significant decrease in mortality at 28 days and a longer duration of hospital stay [7]. This mortality benefit was more evident in patients on mechanical ventilation (29.3% vs. 41.4%; rate ratio, 0.64; 95% confidence interval, 0.51 to 0.81).

Methylprednisolone is a corticosteroid with a relatively short half-life, with a potent anti-inflammatory action and good lung penetration. The evidence of its benefit in the treatment of ARDS is a reduction in mechanical ventilation time and mortality [8,9].

## Case presentation

Herein we describe a 63-year-old patient with modifiable cardiovascular risk factors, dyslipidemia, obesity, hypertension, osteoarticular pathology, and depression, admitted to a district hospital between April 10th and 13th of 2020 with severe ARDS, intubated and ventilated with clinical worsening. He was initiated on a course of antibiotics with no history of the previous administration of remdesivir or tocilizumab. On April 13th, due to clinical aggravation, the Intensive Care Service of Coimbra University Hospital Center (CHUC) was contacted for rescue therapy under ECMO, and the patient was transported to the reference center at CHUC.

The patient was under ECMO between the 1st and 22nd day of hospitalization. During this period, positioning was performed in the prone position, with a slight response. ECMO was suspended due to hemorrhagic complications in the left anterolateral chest wall, where the patient was with an ECMO flow of 5 L/min, a sweep gas flow of 2 L/min, a FiO_2_ at the ventilator of 0.4, PEEP (Positive End-Expiratory Pressure) 8 cmH_2_O with a tidal volume of 290 ml. After discontinuation of ECMO, the patient was given fentanyl analgesic, was sedated with propofol and midazolam, and was given cisatracurium besylate for neuromuscular blockade. In invasive mechanical ventilation under protective lung ventilation in pressure-controlled mode with FiO_2_ 0.8, PEEP 10 cmH_2_O, respiratory rate of 24 breaths per minute, pressure above PEEP 26 cmH_2_O, tidal volume of 6 ml/kg ideal weight, driving pressure > 15 cmH_2_O with 90% oxygen saturation, without the benefit of prone position or recruitment maneuvers.Flexible bronchoscopy was performed on the 23rd day, which revealed moderate bronchial inflammation and a moderate amount of mucoid secretions. The microbiological examination of the bronchial aspirate was found negative. Corticosteroids were started on the 24th day with an initial intravenous bolus regimen of 250 mg methylprednisolone, followed by 40 mg every six hours for five days and slow weaning. The corticosteroid was suspended on the 39th day. The patient was submitted to tracheotomy on the 29th day of orotracheal intubation, corresponding to the second day of the corticotherapy cycle.

**Figure 1:**
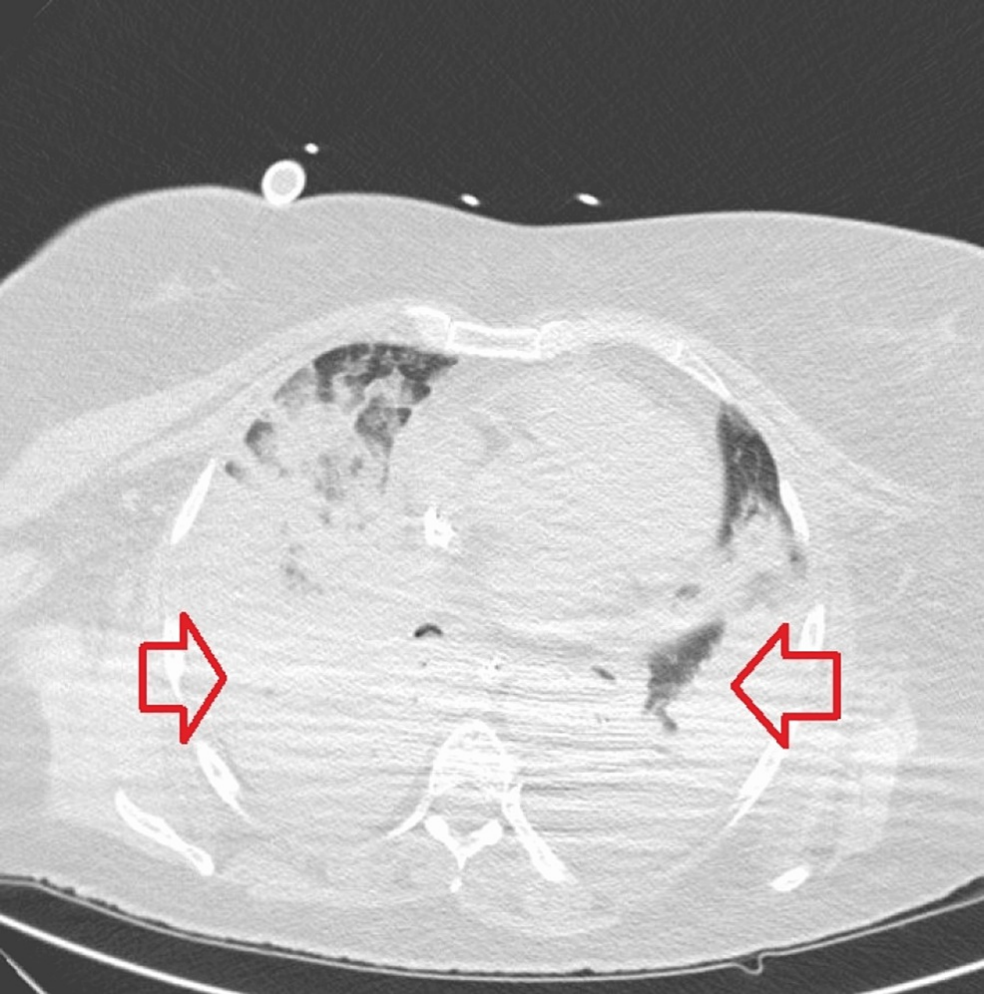
Thoracic CT scan (axial view) on the first day of hospitalization The red arrows show consolidation under venovenous extracorporeal membrane oxygenation

Figure 1 shows the axial computed tomography (CT) scan of the chest performed on the first day of hospitalization. The image revealed extensive parenchymal consolidations with bilateral air bronchogram in all lung lobes, predominantly in the posterior segments, a small volume of airy parenchyma, and evidence of ground-glass appearance densification areas at this level.

**Figure 2:**
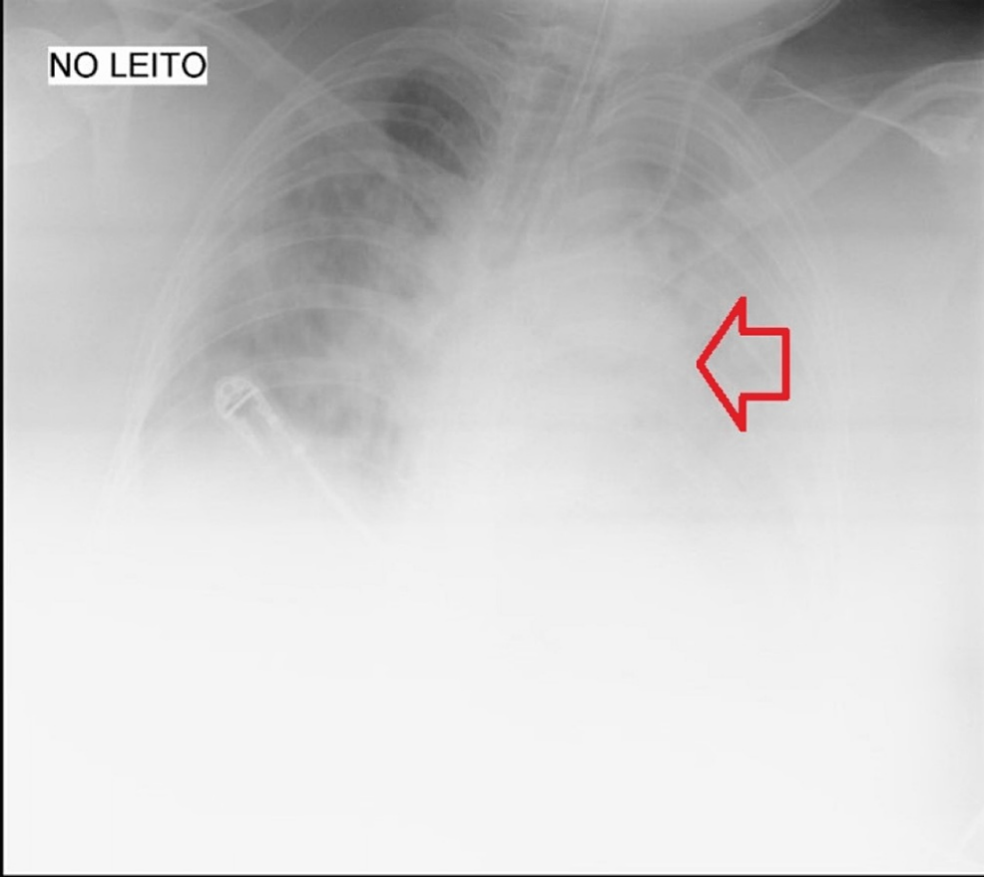
Chest radiography of the day before the start of corticosteroid therapy The red arrow shows opacities

Figure 2 corresponds to the chest radiograph on the day before the start of corticosteroid therapy (23rd day of hospitalization) in which complete opacity of both lung fields with slight sparing of the right apex is observed.

**Figure 3:**
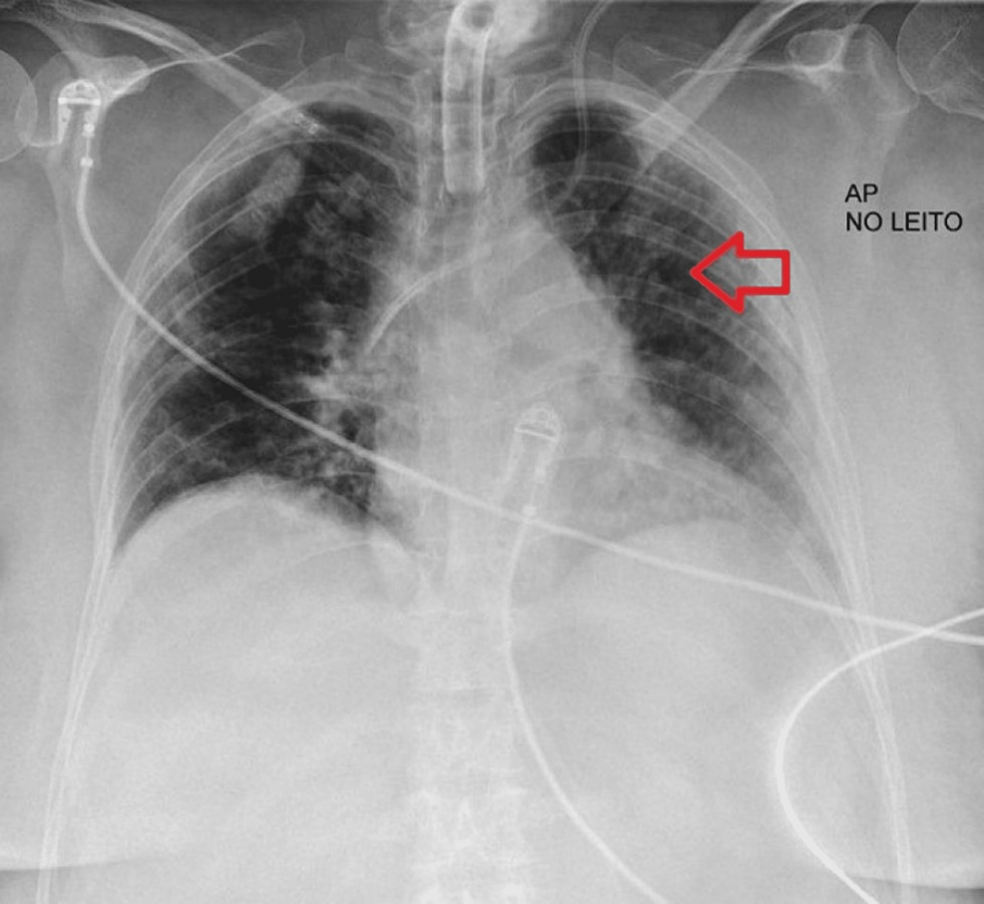
Chest radiograph obtained on the fifth day of methylprednisolone administration The image shows improvement of lung opacities (red arrow)

**Figure 4:**
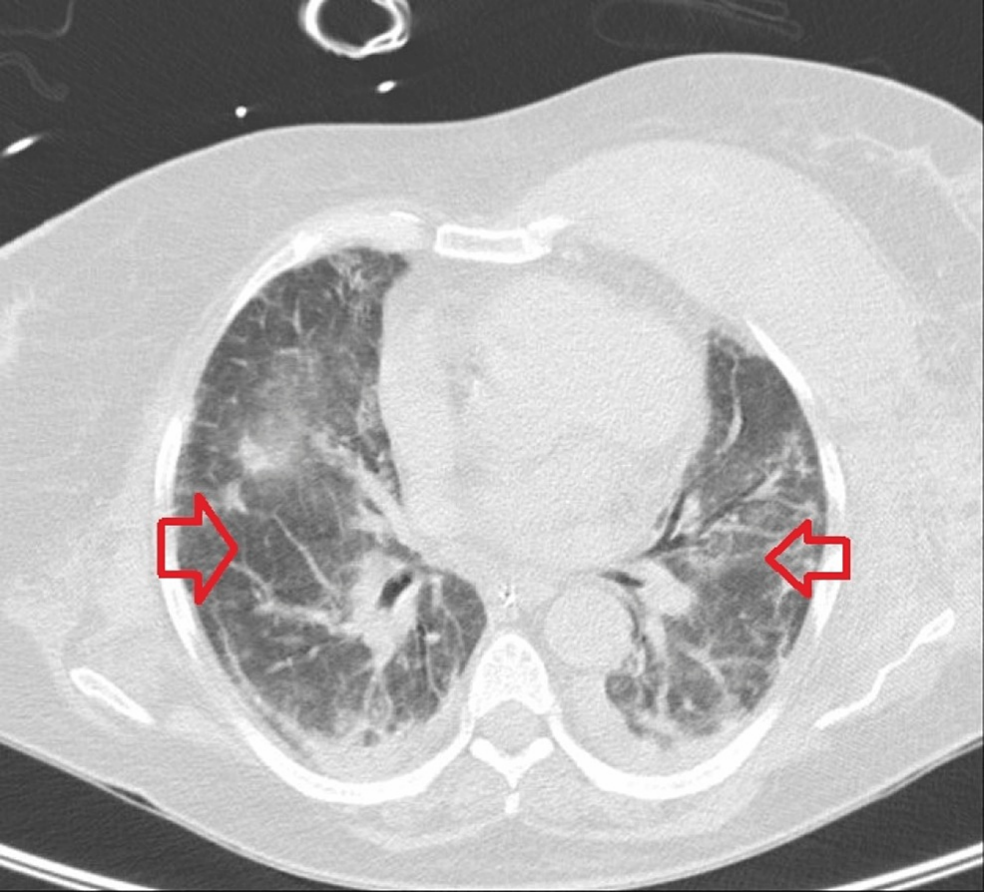
CT scan (axial view) corresponding to the sixth day of methylprednisolone administration The image shows an improvement of pulmonary condensation (red arrow)

Figure 3 corresponds to the fifth day of corticosteroid therapy and presents a reticulonodular pattern with peripheral predominance. Figure 4 refers to the chest CT performed on the 30th day of hospitalization and shows extensive areas of ground-glass densification associated with the thickening of the interlobular septa, predominantly in the upper lobes, with discrete foci of consolidation scattered bilaterally.

Interleukin 6 (IL-6) values ​​were 5.14 pg/ml and 10.41 pg/ml on the 26th and 33rd day of hospitalization, respectively, with an ascending profile after five days of corticosteroid administration. Significant improvement in C-reactive protein (CRP) values ​​(Table 1) and elevation of hepatic enzymology due to drug toxicity were observed. A slight improvement was observed in ferritin and D-Dimer levels.

**Table 1:** Analytical evolution between the 24th and 29th day of hospitalization D – Day; CRP – C-reactive Protein; AST – Aspartate transferase; ALT – Alanine transaminase

ANALYTICALLY	Lymphocytes (x10^9/L)	CRP (mg/dL)	AST/ALT (U/L)	Ferritin (ng/ml)	D-Dimers (ng/mL)
D1 corticosteroid	1.41	25.1	87/94	4234	2619
D5 corticosteroid	1.73	1.43	628/589	3310	2104

Given the improvement in gas exchange, it was possible to suspend myorelaxants, reduce sedatives and analgesics and wean from ventilation. On the 29th day of hospitalization (fifth day of corticosteroid therapy): SatO2 was 94-96% in invasive mechanical ventilation, pressure support ventilation was with FiO2 0.35, PEEP 6 cmH20, PS 17 cmH2O, and tidal volume of 450 - 530 mL.

The patient was discharged to the ICU of the Hospital of origin on May 26th, with a negative SARS-CoV-2 test, under spontaneous ventilation with oxygen therapy at 1L/min, PaO2/FiO2 350 mmHg, with hemodynamic stability and Glasgow coma scale score of 15. The patient was discharged to home after a prolonged stay in a rehabilitation centre.

## Discussion

In the case described, there seems to be a favorable association between starting high-dose corticosteroids (methylprednisolone) and clinical and radiological improvement in this patient with stage III severe SARS-CoV-2 pneumonia. In this case, bronchoscopy made it possible to exclude other causes that could justify the clinical worsening, such as bronchial obstruction, the presence of abundant purulent secretions, or nosocomial respiratory infection that contraindicated corticosteroid therapy.

The preliminary results of the Recovery study seem to demonstrate clinical benefit resulting from the use of dexamethasone only in the group of more severe patients with COVID-19. The potential benefit of the immunomodulatory effect of dexamethasone is its suppressive role in this inflammatory cycle (mediators generate more lung cell injury, which in turn generates more inflammatory stimulus) [10]. A decrease in pro-inflammatory cytokines, corresponding to the adaptive immune response, would be expected, which did not occur with the increase in IL-6. The benefit is more likely to be from the modulated acute-phase reactants and the innate immune response [11]. The failure of corticosteroid therapy described in less severe cases of COVID-19 can be explained, on the one hand, by the non-existence of a treatable hyperinflammatory state and on the other hand, by the unwanted effect of corticosteroids in the suspension of the natural antiviral immune response.

Dexa-ARDS is a randomized, multicenter study that evaluated the effects of dexamethasone in patients with moderate to severe ARDS. The authors observed a decrease in mortality, shorter duration of mechanical ventilation, length of stay in the ICU and in hospital in the corticotherapy group [12]. From a pharmacological point of view, dexamethasone is a strict glucocorticoid, so it does not present the side effects of mineralocorticoids (hypernatremia, volume overload), and it has greater anti-inflammatory potency and a longer half-life [13].

Regarding the dosage, there is doubt about the therapeutic regimen of corticosteroid therapy, but with some evidence of benefit at higher doses in patients with COVID-19. The dosage recommendation for methylprednisolone is the use of a low to moderate dose, ≤ 0.5 to 1 mg/kg/day [14,15]. The regimen chosen for this patient was, however, 2 mg/kg/day, justifying higher doses are used due to the hyperinflammatory state presented, the significant pulmonary compromise, and the unfavorable clinical evolution.

No adverse effects of corticosteroid use were observed in the case described, in addition to the probable contribution to the severe myopathy presented. However, there is a clinical-laboratory dissociation since the immunomodulatory effect of corticosteroid therapy on hypercytokinemia was not evident given the effect of downregulation of the cell-mediated immune response. Also, it may have other effects/mechanisms of action not yet known, in addition to its positive effect on acute phase reactants such as CRP [16].

In summary, corticosteroid therapy is important in the treatment of severe pulmonary disease caused by SARS-CoV-2, particularly in selected cases of the disease in the hyperinflammatory phase, with a potential favorable impact on the clinical course and prognosis of these patients. Current scientific evidence is still inconsistent, and further studies are needed to fully clarify the role of high-dose corticosteroid therapy in critically ill patients with severe SARS-CoV-2 pneumonia.

## Conclusions

This case represents the complexity of the critical patient with the need for ECMO and taking a step forward in fully understanding the role of corticosteroids in critically ill patients. The most common complication of ECMO is bleeding at the insertion of the cannulae or at any anatomical point, so it is important to monitor and assess for signs of bleeding. Performing a bronchoscopy is important to exclude superimposed bacterial or fungal infection and structural injury, which may facilitate the introduction of immunomodulatory therapy. We can conclude that high-dose corticosteroids in the late phase of the disease in selected cases with a hyper-inflammatory stage of the disease would be considered as an option. Dexamethasone, due to its anti-inflammatory characteristics and longer duration of action, could be the corticosteroid of choice in the hyper-inflammatory state.
